# Effects of the Mixing Process on the Rheological Properties of Waste PET-Modified Bitumen

**DOI:** 10.3390/ma16237271

**Published:** 2023-11-22

**Authors:** Grzegorz Mazurek, Przemysław Buczyński, Marek Iwański, Marcin Podsiadło, Przemysław Pypeć, Artur Kowalczyk

**Affiliations:** 1 Department of Civil Engineering and Architecture, Kielce University of Technology, Al. Tysiąclecia Państwa Polskiego 7, 25-314 Kielce, Poland; p.buczynski@tu.kielce.pl (P.B.); miwanski@tu.kielce.pl (M.I.); mpodsiadlo@tu.kielce.pl (M.P.); 2 TRAKT S.A., Szczukowskie Górki 1, 26-065 Piekoszów, Poland; przemyslaw.pypec@trakt.kielce.pl (P.P.); artur.kowalczyk@trakt.kielce.pl (A.K.)

**Keywords:** waste polymers, bitumen modification, statistical modelling, rheological properties

## Abstract

This paper analyses the key findings of a study devoted to PET-modified bitumen. The research program was run according to the D-optimal experimental plan based on a factorial design. Five factors, i.e., the type of polymer (source), the type of bitumen (qualitative factors), PET amount, mixing rate, and mixing temperature (quantitative factors), controlled the bitumen–polymer mixing process. The experiment included a series of determinations of bitumen’s rheological characteristics obtained by MSCR (Jnr, R) and G*/sin(δ) at 50 °C, 60 °C, and 70 °C. The low-temperature properties of the composite (critical temperature) were evaluated using a BBR test. The findings showed that bitumen modification with PET primarily reduced the creep susceptibility of the bituminous–polymer mixture. The low-temperature characteristics of the modified bitumen played a secondary but essential role. The amount of polymer and the mixing rate interacted with the temperature, significantly reducing the stiffness of the composite, while the type and amount of bitumen had a substantial effect on the results obtained in the BBR test. It is worth noting that when combining bitumen and plastomer, special attention should be paid to ensuring a high level of homogeneity of the mixture by controlling the parameters of the mixing process accordingly. The tests and analyses provided crucial models (GLM), which allowed for the prediction of the plastomer-modified bitumen’s low- and high-temperature properties. The resulting relationships between factors and the identification of their impact on the bitumen properties enable a better understanding of the process of bitumen modification with PET. The conclusions presented here serve as a basis for future optimisation of the modified bitumen composition. The performed studies indicate that the use of >3% plastomer in bitumen 70/100 allows for a reduction in its susceptibility (MSCR) to below 0.5 kPa^−1^, making it suitable for bituminous mixtures for high-traffic roads. No significant increase in critical temperature (BBR) was observed.

## 1. Introduction

Bitumen binder, a by-product of petroleum distillation, is a thermoplastic material that determines the durability of bituminous mixtures [1], whose rheological properties depend mainly on the characteristics of the bitumen used [2]. Most pavement distresses are caused by high and low temperatures, cyclic loading, and bitumen ageing. The most well-known way to improve the rheological and functional properties of bitumen for a high-quality binder is to add a polymer into its matrix [2,3]. Elastomers, e.g., styrene–butadiene–styrene (SBS), are the most commonly used [4] and effective polymers for bitumen modification, as confirmed by numerous studies. SBS-modified bitumen in bituminous mixtures improves the mix fatigue life and resistance to moisture and permanent deformation [5,6,7]. The molecular structure of SBS allows a cross-linked network to be formed via the process of polymer swelling and further absorption of the bitumen maltene fraction. Rigid polystyrene blocks in SBS are responsible for bitumen stiffness, while the polybutadiene blocks provide flexibility. In summary, the use of elastomers extends the viscoelastic range of the base bitumen, ensuring its high performance at low temperatures [8,9].

On the other hand, polymers from the plastomer group are a different class of bitumen modifiers. According to the literature, the primary effect of their inclusion in bitumen is an improved resistance to permanent deformation [10]. Thermoplastics are widely used in various industries (chemical, textile, food, etc.) due to their hardness and chemical inertness [11]. They are also incorporated as an additive into bituminous mixtures for bitumen modification. As highlighted by some researchers, the softening point of plastomers should be below the component mixing temperature, which is typically between 160 and 170 °C [12,13]. The use of a hard plastomer raises the bitumen softening point, reduces stability and rut resistance, and lowers the bituminous mix’s ability to withstand low temperatures. 

As of 2018, many countries producing significant amounts of plastomers (including PET) have imposed some restrictions on their import. As a result, special attention is now paid to plastic recycling. Using waste materials in bituminous mixtures allows for benefits such as reducing mix production costs and harnessing the potential of accumulated waste [14].

Different ways of recycling plastomers may include collection, sorting, and shredding. Chemical processes such as pyrolysis and gasification are also available. No less popular, however, is the mechanical method, which provides the material in the form of pellets of various particle sizes [15]. The final properties of polymer-modified bitumen depend on the type and properties of the added polymer and bitumen, the proportions used, and, importantly, the mixing process [16]. The polymer reactivity and chemical structure affect compatibility with bitumen, ultimately affecting the blend quality [17]. During bitumen modification with plastomers, segregation of the components may occur due to the high molecular weight and polarity of the polymer or insufficient maltene fraction in the bitumen [18]. The mixing process can also be affected by high mixing temperatures maintained for long periods, which causes additional bitumen ageing due to maltene fraction degradation or polymer oxidation [6]. As a result, the stability rapidly decreases when the blend is stored. One of the solutions to the low storage stability is to reduce the proportion of non-polar polymer chains by using, for example, butyl acrylate or reactive polymers [19,20]. As a result of the ageing-related higher mixing temperature as compared to ordinary elastomers, modification with plastomers provides additional polar groups and hence an improved bitumen–polymer compatibility [21]. Thus, the technological modification process is a crucial factor to account for during bitumen and plastomer homogenisation, as has been increasingly highlighted in publications. Naderi et al. [22] pointed out the complexity of the kinetics of polymer–bitumen mixing, which are significantly influenced by the polymer content, compatibilizer amount, mixing speed, temperature, and time, as well as the geometry of the mixer. The same conclusions were reported by researchers studying bitumen–SBS compatibility [23]. The ultimate properties of plastomer-modified bitumen will, therefore, depend on the type of bitumen (chemical composition) and available raw materials in the form of plastomers. Therefore, successful modification with plastomers is an iterative process by “trial and error”. 

Among a wide variety of plastomers on the market, there are several available in large quantities, e.g., polypropylene (PP), polyethylene terephthalate (PET), polyvinyl chloride (PVC), and high-density polyethylene (HDPE). In the present study, special attention was paid to PET-type plastomerx. PET is a polymer from which most water bottles and food packaging are made. Its beneficial effects of increasing the bitumen stiffness at high temperatures and thus its impact on the resistance to permanent deformation of bituminous mixtures have been confirmed in a few publications [24,25]. The wider use of PET is limited by its price. The price of waste PET is almost half that of SBS elastomer. Its base solubility is comparable to asphaltenes, so the mixing process will be significantly hindered compared to SBS. In addition, due to its lower solubility, PET will not produce the same cross-linked network as the elastomer under the same manufacturing conditions. However, paper [26] indicates that dosing as little as 2% PET significantly contributes to reducing mix deformation. On the other hand, a combination with a small amount of SBS (about 3%) results in the modified bitumen having properties comparable to that modified with SBS alone. The presence of a small amount of elastomer will compensate for the deficiency in elasticity characterised by bitumen ductility [26]. An excessive PET content reduces bitumen ductility. An increase in the PET content improves the stiffness and viscosity of bitumen and bituminous mixes [27,28]. Studies on PET implementation showed no evident mix stability losses or adverse impacts on the BBR critical temperature when the PET content was up to 6% [29]. 

PET has a high softening point, which may make its homogenisation with bitumen difficult compared to other plastomers [2]. This may also be due to its chemical inertness and high stiffness. However, on this topic, the opinions of researchers vary because the available comparisons refer to bitumen–polymer composites obtained in processes with different configurations of mixing parameters and using PET from various sources [30]. In the case of PET, the major factor that will significantly increase the homogeneity of the bitumen–plastomer mixture is the level of feed shredding. The literature reports adding PET into bitumen by incorporating > 1 mm flakes into bitumen mixtures [31,32] or incorporating it as a material with a high shredding level (<1.18 mm) added directly to the bitumen [33,34]. In the latter case, the plastomer dosage is usually up to 6% by weight of bitumen [35]. When using the second solution, much evidence indicates that the shredding effect is crucial for increasing the elasticity of the binder and the mix [36], as are factors such as the PET origin, contamination, and the chemical composition [30]. These parameters determine the quality of the mixture. Therefore, the mixing process is a critical issue, and a typical selection of mixing parameters in one case may need to be confirmed in experiments performed by researchers using different plastomers. Nevertheless, some aspects and conclusions can be generalised using an experimental design with many relevant factors.

It should be noted that the literature methods do not provide any information on the impact of the mixing process effects and usually consist of certain adopted values of mixing process factors without information on how their selection was determined. The results of the effectiveness of bitumen modification with a modifier are evaluated on the basis of the optimal configuration for SBS application in SBS-modified bitumen. Considering the different polarity and solubility of PET, bitumen modification with PET cannot be compared in the same way to bitumen modified with SBS. Therefore, without identifying the actual effects of the mixing process factors, it is impossible to take a position on the further strategy of modifying bitumen with PET. To the best of the authors’ knowledge, very few comprehensive studies on PET-modified bitumen focusing on the mixing process with different plastomers and bitumens have been published so far. PET waste management is one of the biggest environmental challenges today, and promising test results should encourage further research [34,37]. Incorporating waste PET into bituminous mixtures has already attracted considerable interest [38]. In line with this trend, the present article provides information on the method of controlling the process of bitumen modification with PET, taking into account several variables/factors and rheological effects. The innovation of this operation was to perform mixing in a colloid mill rather than in a laboratory mixer. Thus, the analytical results are much closer to those obtained in real-life conditions. In addition, the identified relationships and their scale allow for effective optimisation of the PET and bitumen mixing process.

## 2. Materials and Methods

### 2.1. Bitumen

In the initial research phase, the bitumen to be modified with the chosen plastomers was selected and comprehensively evaluated [39]. Based on the conclusions from previous studies [34,40], two different types of bitumen, 50/70 (Orlen, Poland) and 70/100 (Orlen, Poland), were chosen and subjected to basic rheological tests. The choice was defined by the need to reproduce different rheological states of the bitumen. The test results and 95% confidence intervals are compiled in Table 1. 

### 2.2. Waste Plastomers

The properties of the bitumen were modified by adding PET (polyethylene terephthalate) from two different suppliers. The plastomer used was a thermoplastic material with a crystalline structure. According to the suppliers’ specifications, the PET had a maximum melting temperature of Tp = 256 °C and a maximum glass transition temperature of 75 °C. 

**Figure 1:**
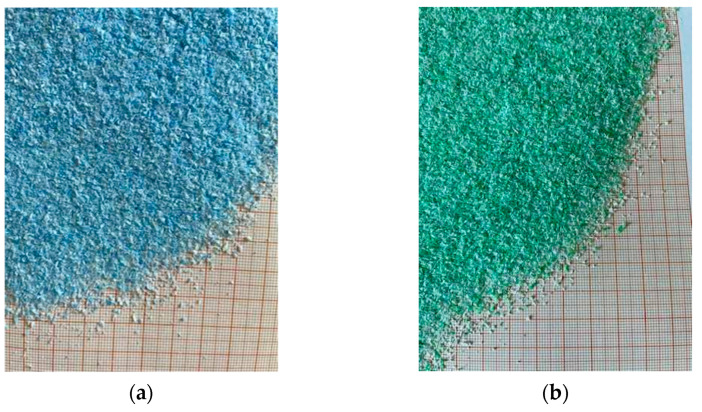
Test polymers; (**a**) supplier 1 (SC1); (**b**) supplier 2 (SC2).

The polymers were pulverized to maximise PET shredding. The samples shown in Figure 1 were then subjected to a granulation process so that the final polymer material had a grain size of <1 mm. The PET suppliers are uniquely identifiable by the plastomer colour resulting from the sorting process. Samples from supplier 1 were labelled SC1 (blue), and those from the second supplier were labelled SC2 (green).

### 2.3. Experimental Plan

The experimental plan required a customised approach. The sampling scheme was important for the prediction of dependent variables. It also influenced the formulation of conclusions. The starting topology of the plan included different values of bivariate and trivariate input quantities. These types of experimental plans combine fractional factorial design types 2^(k−p)^ and 3^(k−p)^ and were classified by Connor and Young for the US National Bureau of Standards [46]. The experimental domain, composed of the independent (controlled) variables listed in Table 2, served as the initial settings for the mixing process.

Quantitative variables were entered at three levels while qualitative variables were at two levels. Additional coding was required for qualitative variables. This procedure was necessary to build a regression model determined based on the widely described generalised linear model (GLM) algorithm [47]. Accordingly, the type of polymer from supplier CS1 was given a code value of 0, while SC2 was given a value of 1. Similarly, in the case of the bitumen type, bitumen 50/70 was given a code value of 0, while bitumen 70/100 was given a value of 1. However, as the entire formula design would require 54 combinations, which would be inefficient under production conditions, the experimental design was altered to consider the adopted final mathematical model employed in subsequent calculations. The initial mathematical model was in the form of a polynomial function (1):

(1) $$y=b_{0}+b_{1}x_{1}+\cdots b_{k}x_{k}+b_{12}x_{1}x_{2}+\cdots +b_{k-1.k}x_{k-1}x_{k}+\cdots +b_{11}x_{1}^{2}+...+b_{kk}x_{k}^{2}$$

where b_k_—experimental coefficients, x_k_—variables, y—dependent variable. Model (1) required some modifications, as two qualitative controlled variables could not be described by the quadratic terms of the equation. Therefore, for these variables, only the effect of the individual influence of a given variable on the dependent variable and their interactions with the other variables were included in the model. The modified model was further evaluated using D-optimal algorithms. Their task was to maximise the determinant of the X^T^X information matrix [48]. This was a key measure to minimise the volume of the total confidence set of all regression coefficients and increase the efficiency of inference about model parameters. In addition, the G-optimality criterion [49] was used (2):

G-optimality = 100 × square root(p/N)^1/2^/Σ_M_ (2)

where p—the number of factor effects in the design, N—the number of required layouts, Σ_M_—the maximum standard deviation of the predicted dependent variable value, with all proposed points considered. The G-optimality criterion minimises the largest value of the standard deviation of the determinable response surface (of the dependent variable determined from the approximating function). As a result, to meet the conditions for maximising the value of the G-optimality criterion, an experimental design containing 32 combinations was constructed, where 16 cases involved SC1 polymer while the remaining 16 used SC2. Each dependent feature was replicated at least twice, depending on the precision assigned by the standard requirements. It was very important to randomise the execution order of each combination in order to minimise the bias from the operator’s precision in the execution of successive mixtures. Therefore, the order of execution of bitumen and plastomer mixtures was implemented in a coded form after randomisation. The experimental domain used in the study is shown in Table 3.

### 2.4. Sample Preparation

The test samples were mixed according to the plan adopted (Table 3) in a colloidal mixer under in situ conditions to obtain mixing conditions corresponding to those at the manufacturing plant. Based on the experiments presented in papers [39,40], a constant mixing time of 45 min was established for all formulations. A 15 min pre-mixing period was introduced before the main mixing period. After the mixing process, the material was left to cool. The tests started after a minimum of 24 h.

### 2.5. Testing the Low-Temperature Creep of Bitumen (BBR)

The Superpave system uses a bending beam rheometer (BBR) to study the behaviour of bitumen at low temperatures. The test is performed according to EN 14771 [45]. To test the binder using the ATS (Rolling Meadows, IL, USA) BBR rheometer, beams made of a plastomer-modified mixture with dimensions of 125/12.5/6.25 mm were prepared from the original RTFO-aged and PAV-aged binders following the SHRP procedure. The binders were tested at −10 °C, −18 °C, and −26 °C. The beams were initially loaded with a force of 30 ± 5 mN. After that, a constant test load of 980 ± 50 mN was applied in the middle of the beam span. An extended unloading period was used. The total test time was 480 s, split into equal loading and unloading periods of 240 s. During the test, the beam deflection was continuously recorded to calculate the creep stiffness modulus (Sm) and the change in creep stiffness (m) after a loading time of 60 s. As a result, the minimum critical temperature T_crit(Sm)_ was determined using the BBR test at Sm = 300 MPa and T_crit(m_) at m = 0.3.

### 2.6. Testing the High-Temperature Creep of Bitumen (MSCR)

A multi-stress creep recovery (MSCR) test was carried out according to EN 16659 [44] using a Discovery Hybrid Rheometer DHR-2, Hanover, MD, USA (DSR). It is a measuring system of parallel plates 25 mm in diameter with a gap of 1 mm. The test procedure involves repeatedly applying a load lasting 1 s to an RTFO-aged bitumen sample at constant stress. Each loading is followed by a recovery period of 9 s. A single creep recovery cycle lasting 10 s is repeated 10 times at a stress of 0.1 kPa, then further increased to 3.2 kPa. The measurement temperatures were 50 °C, 60 °C, and 70 °C.

### 2.7. Rutting Potential G*/sin(δ) in a DSR

Dynamic tests were performed according to the requirements of EN 14770 [50] using DSR. G*/sin(δ) measures the stiffness of the binder at high summer temperatures. Obtaining a high value of the G* modulus and a low value of phase angle δ is advisable to minimise permanent deformations. Such a relationship allows for a highly elastic response of the binder. The values depend largely on the temperature and frequency of the applied load. The frequency value of 1.96 Hz at which the G*/sin(δ) test was performed corresponded to an approximately 80 km/h vehicle speed, as per the provisions of the SUPERPAVE program (SHRP) [51]. The study used a measuring system of parallel plates with a diameter of 25 mm and a gap of 1 mm. The measurement temperatures were 50 °C, 60 °C, and 70 °C. In Poland, the recommended value of G*/sin(δ) is ≥1.0 kPa for unaged bitumen at the maximum pavement temperature.

## 3. Results

### 3.1. Evaluation of the Effect of Process Factors on Bitumen Properties

The first step in studying the influence of process factors on the properties of PET-modified bitumen was an analysis of variance (ANOVA), performed to determine the variation within the population that allows for searching for the parameters of model (1). In other words, an ANOVA determined whether there was at least one pair of results in the studied group with a significant difference at the assumed 95% probability level. Eleven dependent variables were used. The results are shown in Table 4.

The purpose of evaluating the complete model in Table 4 was to determine whether the choice of parameters was appropriate regarding their sensitivity to changing levels of controlled factors. The results in Table 4 indicate that at least one controlled factor in Table 2 significantly affected the dependent variable, providing a solid basis for finding a regression model. In other words, each factor of the mixing process causes a certain change in the bitumen characteristics shown in Table 4. However, the independent result of the influence of controlled factors on each selected dependent variable seems much more interesting. Before performing the main analysis of variance, the homogeneity of variance was assessed using the Levene test [52]. This test, developed in the 1960s, is less sensitive to the assumption of normality. Small sample sizes were used, which complied with standard requirements. As the samples were equinumerous in groups, it was reasonable to use the Levene test. In addition, prior to performing all analyses, outliers whose values were in the spread range defined by three standard deviations were eliminated. The ANOVA results for each characteristic are shown in Table 5.

A significant effect of a given factor of the mixing process is marked as “S”, and the absence of any significant effect as “N”. The ANOVA results in Table 5 confirm the findings presented in Table 4 and allow for some inference. The rotational speed and bitumen type significantly affect the variability of the results of the non-recoverable part of creep compliance, Jnr, and most cases of percent recovery, regardless of the test temperature. Also, the T_crit(m)_ parameter determined in the BBR test indicates dependencies on the two mentioned factors. The type of bitumen caused significant changes in the set of G*/sin(δ) values at 50 °C and 60 °C. For this feature, the amount of polymer turned out to be important. The most significant number of essential factors in the mixing process shaped the variability of T_crit(Sm)._ This feature is responsible for the behaviour of plastomer-modified bitumen at low temperatures. The bitumen type had a significant effect on bitumen properties. The lack of impact of the plastomer content on the Jnr level and the lack of a bitumen–plastomer-type interaction effect on the set of dependent variables were surprising. The analysis of variance revealed a strong effect of the mixing process on the improvement in the mix homogeneity. It can thus be expected that an increased mixing rate prevents the coagulation of plastomer particles, enhancing the interaction strength at the bitumen–plastomer phase boundary resulting from a larger PET–bitumen contact area. The validity of considering the mixing parameter effects was revealed when the BBR test results showed that the mix homogeneity was essential for obtaining a low critical temperature. The analysis of variance indicated a strong relationship between a low critical temperature and the mixing temperature. This is an important finding, as a high mixing temperature that is close to the PET melting temperature affects the increase in PET-modified mix homogeneity. However, the analysis of variance is a linear model with interactions between qualitative factors. Therefore, the suspected multicollinearity between variables and the lack of other interacting factors do not allow for a comprehensive decomposition of existing effects in the dataset. For these reasons, the next step was to perform a PCA.

### 3.2. Principal Component Analysis (PCA)

**Figure 2:**
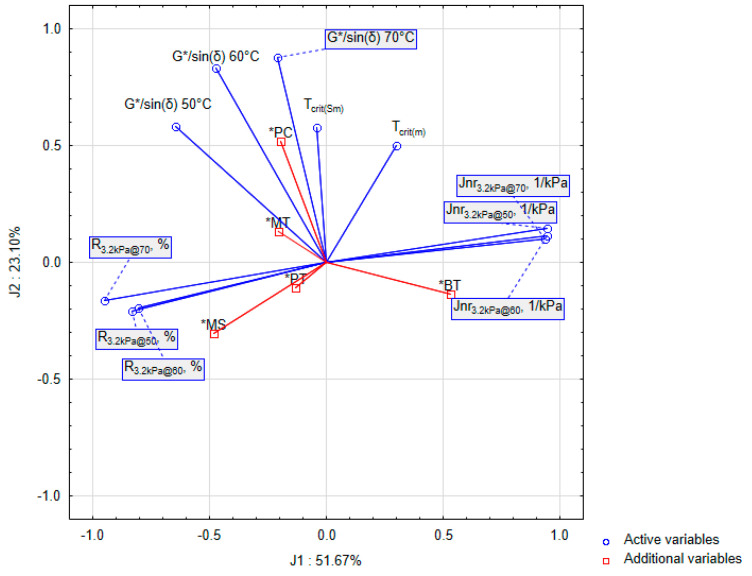
Group variable analysis of the properties of PET-modified bitumen.

In practice, grouping variables is a desirable activity. Of the many clustering methods, a principal component analysis (PCA) is an effective technique for grouping and reducing variables. Its algorithm involves formulating mathematical models in linear equations with an idea similar to ANOVA analysis. It is essentially an orthogonal transformation of the observed results into a new set of uncorrelated variables (new principal components) [53]. In the present study, this approach was used to pre-classify the variables as a new space. The results of the projection of bitumen properties are shown in Figure 2.

The principal component analysis provided new variables (J1, J2) in a reduced number, which were a linear combination of the independent variables participating in the experiment. Standardised bitumen characteristics were used to build a new space and allowed for the detection of structure and general regularities in the current dataset. Using Kaiser’s criterion [54], three significant group variables explaining 86% of the variability of the entire set were established. However, only the first two variables, explaining 75% of the variability, were included in further analysis. The last new grouping variable only contributed 11% to the explanation.

The structure of variable distribution indicates the existence of two regularities. The first, labelled J1, explaining 52% of the variability of the entire set, includes the rheological properties of bitumen describing the behaviour of the modified bitumen at high temperatures. The formation of this variable was influenced by the results from the MSCR test (Jnr. R). The length of the vectors assigned to them indicates that an increase in Jnr correlates strongly with a decrease in R. This conclusion is consistent with the known state of knowledge, since a reduction in bitumen compliance also means a decline in its viscosity and a propensity of the bitumen structure towards a colloidal sol-type state. In establishing the relationships in the set, the effect of changing the measurement temperature was omitted, as the results in Figure 2 strongly correlate within the same characteristic regardless of the temperature type. Marked in red are the values assigned to the levels of controlling factors (independent variables). They did not participate in creating a set of new variables but were dropped into the existing principal component space. This allowed their correlation to be compiled into a new orthogonal result space. Comparing the vectors of dependent and independent variables, it should be concluded that the mixing rate and the type of bitumen are responsible for the change in the properties of modified bitumen at high temperatures. In the case of the Jnr characteristic, its increase was associated with using 70/100 bitumen. In contrast, the increase in mixing speed lowered it, probably due to the better homogeneity of the mixture. This key finding states that more than slow-speed mixing will be needed to ensure the high homogenisation of PET-modified bitumen. Thus, the values of elastic recovery, R, for the cases of mixtures prepared at low mixing speeds were reduced. The direction and level of the mixing temperature vector correlated with the first principal component, J1, suggesting that the applied mixing temperature range is responsible for the decrease in bitumen compliance and the increase in its elastic recovery to a lesser extent than the rotational speed. The modifier content and type had the lowest effect on Jnr and R.

The second component variable, J2, was identified based primarily on the variability of the T_crit(Sm)_ and T_crit(m)_ test results (BBR) and, to a lesser extent, of the G*/sinδ results. The vector assigned to the BBR features was orthogonal to Jnr and R vectors, so searching for the J2 variable was justified. Its contribution to explaining the variability of the entire set was approximately 23%, i.e., half as high as the principal component J1. Therefore, the process of bitumen modification has a much smaller impact on the change in the low-temperature properties of the bitumen compared to its rheological properties at high temperatures. In the case of features T_crit(Sm)_ and T_crit(m)_, the amount of polymer showed the highest correlation with them, suggesting that an increase in the amount of plastomer leads to an increase in the critical temperature. In the case of the G*/sinδ feature, increasing the amount of plastomer stiffens the bitumen. The relationship between T_crit(Sm)_ and G*/sinδ indicates that the increased stiffness of modified bitumen at high temperatures has an adverse effect on the increase in the critical temperature of the modified bitumen (tends to positive values). Therefore, the amount of plastomer requires special attention because, on the one hand, it reduces the bitumen deformation potential. Still, on the other hand, it makes it brittle at low temperatures. With respect to the mixing temperature, its increase contributes to a minor but unfavourable increase in the critical temperature, which may result from the progressive bitumen ageing process during mixing. An inverse relationship was observed between mixing speed and higher-penetration-grade bitumen 70/100. To a small extent, an increase in the mixing speed and the use of a high penetration grade reduced the critical temperature determined from the BBR test, which may be an indirect result of an increased level of mix homogeneity.

### 3.3. Modelling of Bitumen Properties Using a Generalised Linear Model (GLM)

The generalised model (GLM) was used to model the relationship between the characteristics of plastomer-modified bitumen and the mixing process variables. Its syntax allows for the quantitative and qualitative variables to be taken into account. Another notable advantage of GLM models is the inclusion of interaction effects and nonlinear terms in the function. Accordingly, model (1) was adopted as the research object function. Its form was introduced using an identity function binding the vector of independent variable values to the vector of expected values. This is because the distribution of the dependent variable, that is, the bitumen characteristics, had a normal distribution [47]. The generalised form of the adopted model was as follows (3):

(3) g(Y)=X·β+ε

where Y—vector of dependent variable values, X—matrix of independent variable values, ε—random component of residuals, β—vector of model coefficients, g—binding function. The last issue to consider was how to implement the qualitative variables. Accordingly, the qualitative variables were coded on a binary scale of 0–1 and were implemented in the regression model using the so-called “over-parametrised model”. In this situation, a generalised inverse of the X’X matrix is required to determine the vector of model coefficients. Due to the modification of the initial design, the generalised inverse in the case of an incomplete-order matrix was calculated by simply intentionally zeroing the elements in the redundant rows and columns of the matrix [55].

From the fact that there is a high correlation between the results of some bitumen characteristics, depending on the test temperature (Figure 2), modelling results are presented only using selected characteristics such as:

- G*/sin(δ) at 60 °C;
- T_crit(Sm)_ at Sm = 300 MPa;
- Jnr_3.2kPa@60_, kPa^−1^;
- R_3.2kPa@60,_ %.

The optimal set of independent features describing the subsequent dependent variables was selected using the backward stepwise approach. This is a combination of the backwards elimination method and stepwise regression. Such a set may include significant variables with a *p*-value of < 0.05 and other variables selected to obtain the minimum value of the Akaike criterion (AIC) [56] and the minimum value of Pearson’s scaled chi^2^ statistic with respect to model (1). The first variable evaluated was the rutting coefficient G*/sin(δ). The optimal set of predictors is given in Table 6.

**Figure 3:**
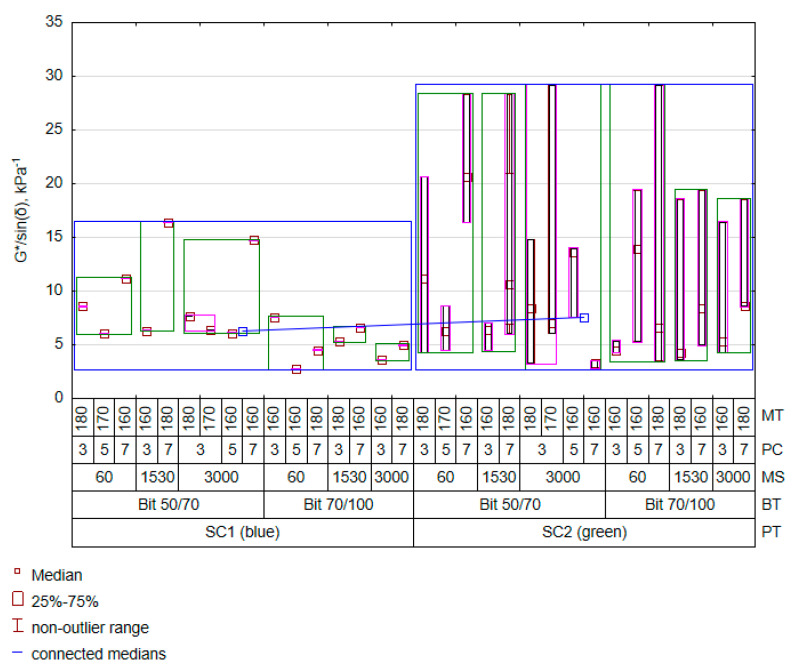
Variation in predicted G*/sin(δ) results.

The Level column represents the adopted reference value related to a given qualitative variable. When searching for a solution using another level of a qualitative variable, the value of this term in the equation will be zero. After the analysis, the final optimal model consisted of 12 parameters. The model explained approximately 52% of the variability in the dataset with an average estimation error of 3.19 kPa. This error is higher than the recommended higher minimum value of 1 kPa. Therefore, the minimum value will probably be obtained at a very low temperature, indicating that the contribution of a given bitumen to the mix deformation resistance will be considerable. The most significant impact was recorded for the plastomer type and the interaction with plastomer content in the mixture. In addition, the interaction between the polymer content and bitumen type also came into play. The number of interactions confirms the partial results given in Figure 2, thereby supporting the statement that the presence and quantity of PET are crucial from the perspective of controlling the G*/sin(δ) value. This is due to the fact that the plastomer stiffness is higher than that of bitumen. The effect of incorporating > 3% plastomer will be similar to that of a fine-grained filler, the only difference being that, to a small extent, the plastomer increases the elasticity, which will affect the elasticity of the mix. In the case of the G*/sin(δ) characteristic, its amount and type increased the high-temperature stiffness, which will have a measurably beneficial effect on the bituminous mixture in the summer season. Despite the chemical inertness of PET and its low solubility, the mentioned parameters of the mixing process play a special role in shaping the structure of PET-modified bitumen. Moreover, the interaction effect between the amount of polymer and the bitumen type suggests certain interactions in the bitumen–PET contact zone. The presented results complement the conclusions drawn from the ANOVA in Table 5. Extended findings, obtained in the context of relevant factors, result from the adopted model that takes into account interaction effects, additionally decomposing the total variability by adding new cognitive elements of the bitumen–plastomer mixing process. A visualisation of the fitting results against the cases defined in the experimental plan (Table 3) is presented in the variability diagram below (Figure 3).

From the model, it follows that the SC2 polymer (green) increases the average value of the G*/sin(δ) parameter. However, soft bitumen 70/100 significantly reduces the stiffness of the modified bitumen compared to bitumen 50/70. Another significant observation is that the G*/sin(δ) level increase is dependent on the PET content increase. Researchers studying other plastomers have reached the same conclusions [57].

The MSCR results complement the assessment of PET’s effects on the properties of bitumen at high temperatures. As in the case of the G*/sin(δ) feature, an attempt was made to model the Jnr value using mixing process factors. A regression model was obtained with the same assumptions to predict the non-recoverable part of compliance Jnr_3.2kPa@60_ at 60 °C, see Table 7.

**Figure 4:**
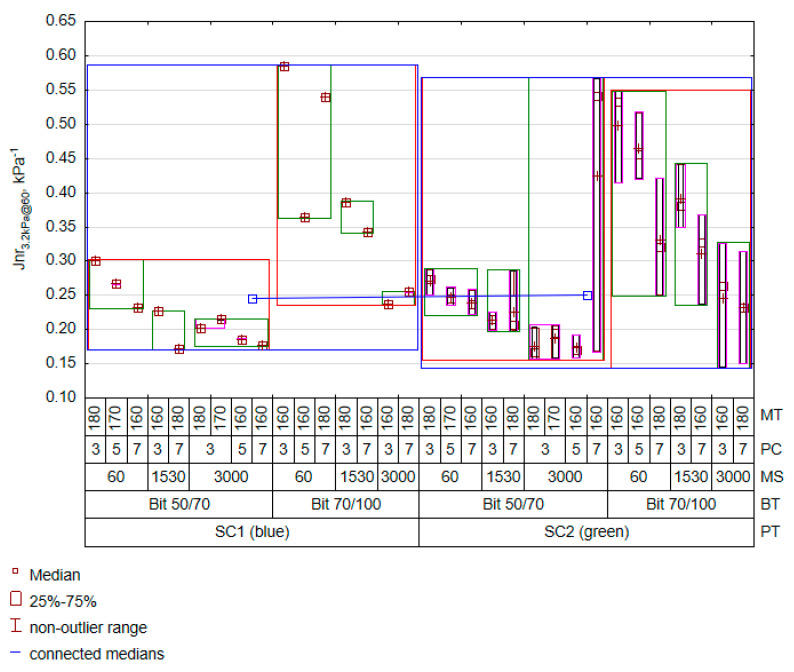
Variation in predicted Jnr_3.2kPa@60_ results.

The creep of bitumen modified at a temperature of 60 °C strongly depended on the rotational speed. The remaining factors were strongly implicated in interactive effects. An interesting observation that emerged from Table 7 was the interaction between the type of bitumen and the amount of plastomer (BT*PC). Compared with G*/sin(δ), its statistical significance for the Jnr_3.2kPa@60_ feature turned out to higher. This means that an increase in the amount of plastomer with a decrease in the penetration grade of the bitumen (bitumen 50/70 was specified as the reference) results in a rapid decrease in bitumen susceptibility, probably leading to an improved resistance of the mixture to permanent deformation. This proves that the resistance to deformation of bitumen with PET in cyclic creep testing is a complex phenomenon. Not accounting for the type of plastomer feature in the model is a symptomatic issue. With a coefficient of determination of R^2^ = 71%, a low estimation error of RMSE = 0.07 kPa^−1^ and a high value of fitting using Pearson’s scaled chi^2^ statistic, it was impossible to determine its influence on the model. Therefore, the level of PET homogenisation with bitumen was much more important than the contribution of plastomer stiffness to the change in the stiffness of the bitumen–plastomer blend. The prediction results corresponding to the cases adopted in the experiment are shown in Figure 4.

The results in Figure 4 show much more clearly that the PET effect causes a specific decrease in compliance, but it remains within the estimation error. The influence of a high rotational speed and the bitumen type on reducing Jnr_3.2kPa@60_ was much more important. Also, despite interaction, a high mixing temperature increases the mixture’s homogenisation level, resulting in reduced creep compliance of the bitumen. Since the PET softening point is high, its homogenisation with bitumen will depend on its value. As in the case of G*/sin(δ, an increase in the amount of plastomer resulted in an increased stiffness of the polymer–bitumen mixture. Another property determined during the MSCR test was the percent recovery R_3.2kPa@60_, which characterises the modified bitumen’s elasticity. The final results of assigning model weights are presented in Table 8.

**Figure 5:**
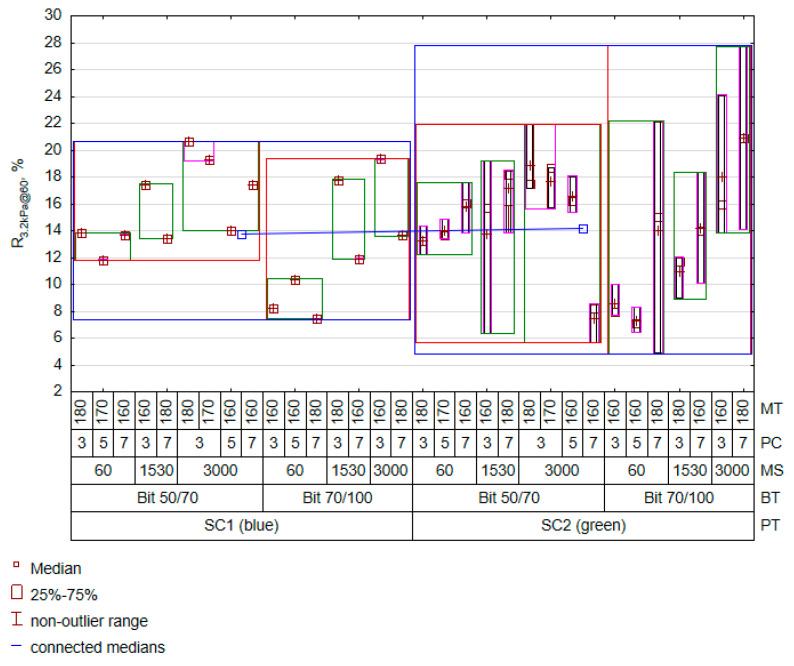
Variation in predicted R_3.2kPa@60_ results.

Table 8 data indicate that the favourable increase in the R value is influenced by the rotational speed in interaction with an increase in the bitumen–plastomer mixing temperature. In this case, the interaction of the plastomer content with the type of bitumen did not cause a significant effect on the value of elastic recovery. The presence of this interaction in the model was solely due to the need to obtain a low estimation error. The amount of polymer present in the interacting relationships almost always results in a consistent decrease in elastic recovery. Thus, the amount of PET increases stiffness but at the same time decreases the elastic deformation capacity of the material, making it an elastic–brittle material with a possibly low LVE range. Unexpectedly, the interaction between the plastomer content and the increasing temperature level favourably increased the R level. The high R^2^ and the low value of the RMSE error suggest that the adopted model properly describes the R variation using the adopted mixing process factors. The predicted value of recovery R against the assumed factor levels is shown in Figure 5.

**Figure 6:**
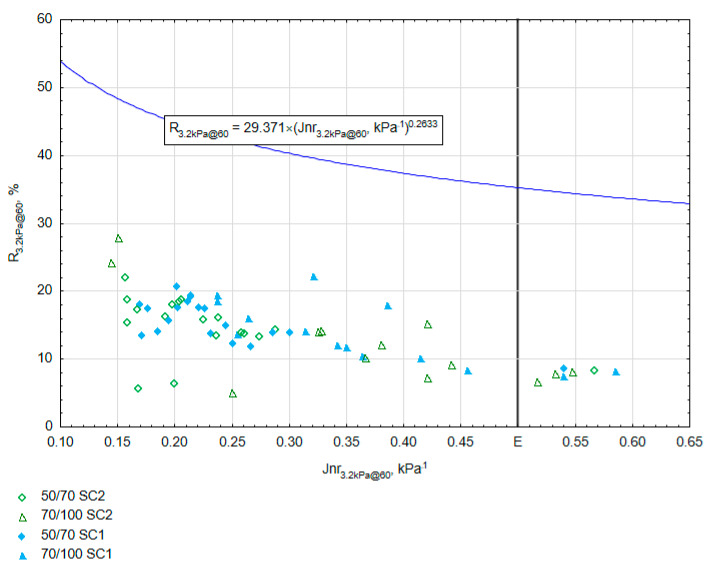
R vs. Jnr in MSCR.

As in the case of the Jnr_3.2kPa@60_ feature, the plastomer type played a secondary role. A much greater variation was observed in relation to changes in rotational speed and plastomer content and the accompanying change in mixing temperature. Thus, the key to obtaining favourable viscoelastic properties is to ensure optimal mixing process parameters, with particular attention paid to mixing temperature, mixing speed, and plastomer content. In light of the conclusions obtained, forming a conclusion about the effectiveness of bitumen modification with PET without performing an experiment is impractical. An analysis of the mixing process should be the starting point for making decisions regarding further modifications with PET. Figure 6 illustrates the scatter of the results for R_3.2kPa@60_ and Jnr_3.2kPa@60_.

While observing the results in Figure 6, please note that almost all the results of PET-modified samples determined at 60 °C obtained compliance, allowing for extremely heavy traffic conditions (class E according to AASHTO M 332) [58]. The average value of Jnr_3.2kPa@60_ for bitumen 50/70 was 2.2 kPa^−1^ and it was 5.7 kPa^−1^ for 70/100, but the elastic recovery value was low—below the solid line on the graph indicating an elastic response. Therefore, the modification of bitumen with PET cannot be considered the same as with SBS elastomer. Please note that the susceptibility of bitumen modified with plastomer SC2 (green) was the lowest (Figure 6) at the level of about 0.15 kPa^−1^. Compared with commercial bitumen PmB 45/80–55 [59], the susceptibility value of the modified bitumen was lower by 0.05 kPa^−1^. The elastic recovery was lower by about 30%, which shows that the modification did not obtain the same level of elasticity as with the use of SBS plastomer. Nevertheless, the elastic recovery R was higher than that obtained for neat bitumen and did not exceed 3% (Table 1). As mentioned, all the results are below the line separating bitumens modified with an acceptable elastomeric polymer. According to the provisions of AASHTO T350 [60], the presence of elastomer-modified bitumen is indicated by an R above the line. However, Morales et al. [61] point out a certain imperfection in the interpretation of the R parameter, which, in a non-obvious way, correlates with the resistance of bitumen mixtures to rutting. Therefore, modification results slightly below the line determined by the threshold function do not indicate that the bitumen in question should be disqualified as modified. However, the response during the MSCR test may be slightly different from other elastomer-modified bitumens. Evaluation of the MSCR results suggests that the effects of PET on bitumen are similar to those of fine filler. The difference between the two is due to the PET elasticity being greater than that of mineral aggregate. If the degree of PET grinding is increased, the bitumen and plastomer are mixed at a higher temperature, and if soft bitumen is used, a higher elastic recovery value can be obtained, probably due to an increase in the bitumen–PET compatibility. However, a high PET content reduces the properties related to the elasticity of the mixture (Figure 6).

The last parameter that characterises PET-modified bitumen is the critical temperature T_crit(Sm)_, calculated for a stiffness of Sm = 300 MPa in the BBR test. This parameter is essential to complement the conclusions on the impact of PET on bitumen. It is vital to ensure that bitumen modification with a polymer does not worsen its properties at low temperatures, which would question the idea of bitumen modification. It should be emphasised that increasing the critical temperature has a considerable limiting effect on the effective viscoelastic range of the modified binder and hence its practical use. The results of modelling using the GLM algorithm are compiled in Table 9.

**Figure 7:**
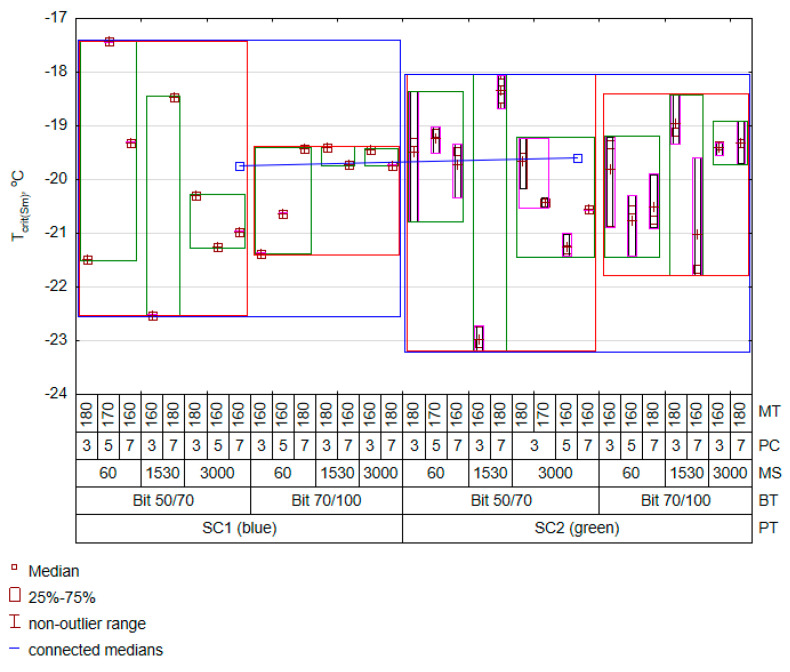
Variation in T_crit(Sm)_ results.

The best fit of the parameters in the adopted model (1) to the experimental results was obtained in the case of the T_crit(Sm)_ feature. Many parameters from the selected optimal set of mixing process parameters considerably impacted the T_crit(Sm)_ results. Most importantly, the nonlinear effect of temperature also played an important role in shaping the critical temperature prediction. For a given (T_crit(Sm)_) feature, the interaction between low-penetration-grade bitumen (the base bitumen for this analysis is 70/100) and plastomer content will increase the T_crit(Sm)_ value. Thus, this relationship is inversely proportional to the advantages of the plastomer content in the context of a decreased susceptibility. Moreover, both the polymer content and the bitumen amount directly influenced the T_crit(Sm)_ property changes. The previous conclusions (Figure 2) are supported by the fact that the increase in the amount of polymer increases the critical temperature of the bitumen. The increase in mixing temperature was correlated with an increase in mix brittleness at low temperatures, probably due to bitumen ageing. The increased mixing speed in the interaction effects suggests an improvement in the characteristics of low-temperature bitumen–polymer mixtures. However, it should be emphasised that the presence of interaction effects indicates the complexity of the process factors’ impact on the low-temperature properties of PET-modified binders. The results of predicting the critical temperature of the polymer–bitumen composites are presented in a variability graph (Figure 7).

An analysis of the results in Figure 7 indicates that the beneficial effect of the SC2 (green) polymer on reducing the bitumen deformability resulted in a slight undesirable increase in critical temperature (the line connecting the medians). An unexpectedly low T_crit(Sm)_ was recorded for 1530 rpm^−1^, 3% plastomer content, and 160 °C, in which bitumen 50/70 was used. This effect, repeated regardless of the source of PET, might be due to the low content of the modifier added to the bitumen at a minimal increase in its ageing level. In this case, the 1530 rpm^−1^ rotation level could evenly distribute the polymer in the bitumen. Regarding the results of Jnr_3.2kPa@60_, it should be stated that the obtained resistance to deformation was moderate. However, regarding the material quality, an increase in the plastomer content resulted in a successive reduction in the bitumen resistance to low temperatures. Similar results were obtained by the authors of study [29], where it was demonstrated that a PET content increase to a level of 6% and enrichment with a compatibilizer does not imply any deterioration of the low-temperature properties of bitumen.

The results above show that it is important to keep in mind the limitations of the proposed model. The parameters were estimated based on the experimental plan given in Table 3. Despite meeting the postulate of normality of residuals for the constructed models, it should be taken into account that the approximation of the values of the dependent variables located at a large distance from the experimental domain may yield contradictory results. Therefore, the present modelling with GLM is valid for minor deviations from the range of the experimental domain.

## 4. Conclusions

This statistical-inference-based study provided a lot of important information about the process of mixing PET with bitumen. The key issue is that generalised conclusions about the effect of PET on bitumen modification cannot be drawn solely based on a modifier from one supplier. This study employed plastomers of two different colours from two sources. From the mixing process observations in the laboratory, it follows that the type of plastomer yielded different effects on the consistency of the mixture during mixing. So, the presence of fillers and pigment in the plastomer will make a huge difference. No comparative studies known to the authors take into account the variability due to the origin of the plastomer. It is widely accepted that contamination can lead to different results for bitumen characteristics, as confirmed by the tests included in this experiment. The level of compatibility of the same plastomer but from a different source was different, as observed in both susceptibility (MSCR) and critical temperature (BBR) tests. An important aspect that supports the representativeness of the results in this study was dealing with the results obtained from samples prepared in a colloid mill. This allowed us to include the effects related to imperfections accompanying the mixing process in the variability of the results.

Also, the consistency of bitumen with different proportions of asphaltene fraction versus maltene fraction repeatedly confirmed significant effects on the rheological properties of the modified bitumen. This finding is also supported by the results of the principal component analysis, according to which about 25% of the variation (Figure 2) cannot be explained by the mixing process factors adopted in the experiment. In this case, the chemical interaction, the mixer’s geometry, and the shredding degree will effectively complement the influence of unexplained variability on the selected dependent characteristics. We should also keep in mind the mixing time, which, for technical reasons, still needs to be evaluated. Its value was assumed based on previously performed exploratory studies based on the Plackett–Burman elimination plan [40]. Nevertheless, given the current state of knowledge, the mixing time factor will be included in subsequent experiments on optimising the mixing process. The confirmed significant impact of PET on bitumen properties allows for the formation of the detailed conclusions below. These detailed conclusions can be the argument for undertaking further implementation measures:

- A principal component analysis of the set variability structure indicated the existence of two dominant factors, explained in approximately 75% by the selected tests on bitumen. The first factor is the bitumen rutting resistance, explained in 52%. The low-temperature properties of PET-modified bitumen can be explained in approximately 23% by the research methods used;
- The vectors assigned to the variables in the PCA and the ANOVA results indicate that a high mixing speed and the bitumen type significantly reduce the compliance of plastomer-modified bitumen. However, the mixing rate (MS) and the use of high-penetration bitumen (PT) minimise this effect;
- A detailed analysis of modelling using GLM indicates that the mixing speed (MS) is a crucial factor for obtaining a low bitumen deformability (MSCR) and a low critical temperature (BBR). This result was probably related to the increase in the homogeneity of the mixture, to which, in the case of PET-modified bitumen, special attention should be paid;
- The amount of plastomer (PC) > 3% was by far the most important factor for ensuring a high G*/sinδ value and a low Jnr, but it had a negative impact on the critical temperature;
- The temperature of mixing (MT) > 1500 rpm^−1^ aids bitumen–PET homogenisation. However, in the case of low-temperature properties, it slightly increased the critical temperature, which might be related to an accelerated ageing process. The low mixing rate does not ensure a satisfactory quality and standard characteristics of the plastomer-modified bitumen;
- The plastomer type (PT) was the least significant factor. However, its impact was complex and involved in numerous interactions;
- The presence of pigment and impurities strongly affects the final quality of the plastomer-modified bitumen;
- Significant bitumen–plastomer interactions suggest different compatibility levels between these components resulting from plastomer solubility differences. Therefore, research should be continued;
- The bitumen type (BT) was the most important factor for low-temperature characteristics and ensuring a high homogenisation of the mixture. The use of lower-viscosity bitumen significantly increased the level of its deformation. To ensure a high resistance of the modified bitumen to deformation, the use of an increased amount of plastomer is required;
- The results of the R–Jnr relationship proved that adding a plastomer allows for the compliance to be reduced to the level of >0.5 kPa^−1^. Therefore, the obtained modified bitumen can be used in extremely heavy traffic conditions.

It should be strongly emphasised that the modification of bitumen with PET does not expand the bitumen viscoelastic range to the level obtained by the modification with SBS. These are two different types of polymers. The lower compatibility between bitumen and PET requires further research, with a focus on the contact phenomena between bitumen and PET. The occurrence of significant interactions in the developed models suggests that the bitumen–PET interaction begins during mixing. Therefore, in the future, studies involving an evaluation of the chemical composition of the plastomer will be implemented. Further optimisation of the mixing process supported by the selection of a suitable compatibilizer will definitely increase the efficiency of PET use. The presented detailed feasibility study of bitumen modification with PET, taking into account the consistency of the input bitumen and the type of plastomer, confirms the possibility of its implementation and is an innovative measure that should be further pursued.

## Figures and Tables

**Table 1:** Paving bitumen results.

Features	Neat Bitumen		Standard
	50/70	70/100	
Penetration at 25 °C, 0.1 mm	60	91.5 ± 3.1	PN-EN 1426 [41]
Softening point T_R&B_, °C	48.6	44.7 ± 0.7	PN-EN 1427 [42]
Fraass breaking point, °C	−15	−13.4 ± 2.0	PN-EN 12593 [43]
Jnr_3.2kPa@60_, kPa^−1^	2.2 ± 0.3	5.7 ± 0.3	EN 16659 [44]
R_3.2kPa@60,_ %	1.7 ± 0.5	0	EN 16659 [44]
T_crit(Sm)_ at Sm = 300 MPa	−16.6 ± 2.0	−16.9 ± 2.0	EN 14771 [45]
T_crit(m)_ at m = 0.3	−15 ± 1.5	−16.2 ± 1.5	EN 14771 [45]

**Table 2:** Input variables (with abbreviations) and their levels in the process and bitumen and plastomer mixing.

Quantitative Variables	Levels	Qualitative Variables	Levels
Mixing speed (MS), rpm^−1^	60, 1530, 3000	Plastomer type (PT)	SC1 (blue). SC2 (green)
Mixing temperature (MT), °C	160, 170, 180	Bitumen type (BT)	35/50 70/100
Plastomer content (PC), % (by bitumen mass)	3, 5, 7	-	-

**Table 3:** Domain of experiment.

Case	Polymer Type (PT)	Bitumen Type (BT)	Mixing Speed rpm^−1^ (MS)	Polymer Content % (PC)	Mixing Temperature °C (MT)
1	SC1	50/70	60	3	180
2	SC1	50/70	60	5	170
3	SC1	50/70	60	7	160
4	SC1	50/70	1530	3	160
5	SC1	50/70	1530	7	180
6	SC1	50/70	3000	3	170
7	SC1	50/70	3000	3	180
8	SC1	50/70	3000	5	160
9	SC1	50/70	3000	7	160
10	SC1	70/100	60	3	160
11	SC1	70/100	60	5	160
12	SC1	70/100	60	7	180
13	SC1	70/100	1530	3	180
14	SC1	70/100	1530	7	160
15	SC1	70/100	3000	3	160
16	SC1	70/100	3000	7	180
17	SC2	50/70	60	3	180
18	SC2	50/70	60	5	170
19	SC2	50/70	60	7	160
20	SC2	50/70	1530	3	160
21	SC2	50/70	1530	7	180
22	SC2	50/70	3000	3	170
23	SC2	50/70	3000	3	180
24	SC2	50/70	3000	5	160
25	SC2	50/70	3000	7	160
26	SC2	70/100	60	3	160
27	SC2	70/100	60	5	160
28	SC2	70/100	60	7	180
29	SC2	70/100	1530	3	180
30	SC2	70/100	1530	7	160
31	SC2	70/100	3000	3	160
32	SC2	70/100	3000	7	180

**Table 4:** ANOVA for the complete model.

Dependent Variable	R^2^	MSE ^1^	F-Stat.	*p*-Value ^2^
G*/sin(δ) 50 °C	0.66	39.66	12.57	0.00000
G*/sin(δ) 60 °C	0.50	13.61	6.43	0.00009
G*/sin(δ) 70 °C	0.36	22.74	3.69	0.00529
T_crit(Sm),_ °C	0.44	1.08	5.08	0.00061
T_crit(m),_ °C	0.45	14.02	5.41	0.00038
Jnr_3.2kPa@50,_ kPa^−1^	0.57	4.51	8.71	0.00000
Jnr_3.2kPa@60,_ kPa^−1^	0.54	0.29	7.67	0.00002
Jnr_3.2kPa@70,_ kPa^−1^	0.53	0.01	7.26	0.00003
R_3.2kPa@50,_ %	0.37	2.84	3.79	0.00454
R_3.2kPa@60,_ %	0.43	0.33	4.83	0.00089
R_3.2kPa@70,_ %	0.34	17.88	3.38	0.00871

^1^ root mean squared error. ^2^ variables with a *p*-value < 0.05 were insignificant.

**Table 5:** ANOVA results (parameterisation with sigma constraints).

Mixing Process Parameter	G*/sin(δ) 50 °C	G*/sin(δ) 60 °C	G*/sin(δ) 70 °C	T_crit(Sm)_, °C	T_crit(m)_, °C	Jnr_3.2kPa@70_, kPa^−1^	Jnr_3.2kPa@60_, kPa^−1^	Jnr_3.2kPa@50_, kPa^−1^	Jnr_3.2kPa@50_, kPa^−1^	R_3.2kPa@60_, %	R_3.2kPa@70_, %	R_3.2kPa@50_, %
MS	N	N	N	N	**S**	**S**	**S**	**S**	**S**	**S**	**S**	**S**
PC	**S**	**S**	**S**	**S**	N	N	N	N	N	N	N	N
MT	N	N	N	**S**	N	N	N	N	N	N	N	N
PT	N	N	N	**S**	N	N	N	N	N	N	N	N
BT	**S**	**S**	N	**S**	**S**	**S**	**S**	**S**	**S**	N	**S**	N
PT*BT	N	N	N	N	N	N	N	N	N	N	N	N

**Table 6:** G*/sin(δ) model parameters.

Independent Variable	Level	Coeff.	*p*-Value ^2^	Scaled Chi^2^ P.	R^2^	RMSE ^1^
Intercept		15.3593	0.313828	1.28	57.2	3.19
BT*MS	70/100	0.0005	0.377883			
BT*MT	70/100	0.0934	0.274619			
PC		−7.4361	0.141744			
PC^2^		0.9539	0.061419			
MS^2^		−0.0001	0.426528			
MT^2^		0.0001	0.668748			
MS*PC		0.0004	0.174998			
BT*PC	70/100	0.9028	0.042943			
PT*MS	SC2 (green)	−0.0008	0.145242			
PT	SC1 (blue)	7.3626	0.001806			
BT	50/70	−18.5021	0.198334			
PT*PC	SC2 (green)	−1.7197	0.000036			

^1^ root mean squared error. ^2^ variables with a *p*-value < 0.05 were insignificant.

**Table 7:** Model parameters for Jnr_3.2kPa@60_.

	Level	Coeff.	*p*-Value ^2^	Scaled Chi^2^ P.	R^2^	RMSE ^1^
Intercept		0.488693	0.000000	1.12	71	0.07
BT*PC	50/70	0.011806	0.017960			
MS*PC	50/70	0.000014	0.000374			
MS		−0.000110	0.000000			
PC*MT	70/100	−0.000162	0.000516			
BT*MS	70/100	0.000032	0.000014			
BT*MT		−0.000957	0.000000			

^1^ root mean squared error. ^2^ variables with a *p*-value < 0.05 were insignificant.

**Table 8:** Model parameters for R_3.2kPa@60_.

Independent Variable	Level	Coeff.	*p*-Value ^2^	Scaled Chi^2^ P.	R^2^	RMSE ^1^
Intercept		6.558793	0.000657	1.14	72	3.4
BT*PC	70/100	−0.303740	0.210987			
BT*MT	70/100	0.023712	0.004149			
MS*MT		0.000029	0.000000			
BT*MS	70/100	−0.001201	0.000690			
MS*PC		−0.000665	0.000317			
PC*MT		0.006114	0.004389			
PT*MT	SC2 (green)	0.002396	0.347372			
BT*PC	70/100	6.558793	0.000657			
PT*MT	70/100	−0.303740	0.210987			

^1^ root mean squared error. ^2^ variables with *p*-value < 0.05 were insignificant.

**Table 9:** Model parameters for T_crit(Sm)_.

	Level	Coeff.	*p*-Value ^2^	Scaled. Chi^2^ P.	R^2^	RMSE ^1^
Intercept		−313.552	0.000076	1.19	78.1	3.48
PT*MT	SC2 (green)	−0.005	0.000147			
BT	50/70	−7.438	0.000000			
BT*PC	70/100	0.358	0.000000			
PC		0.150	0.000732			
MT		3.379	0.000301			
MT^2^		−0.010	0.000411			
BT*MT	70/100	0.034	0.000037			
BT*MS	70/100	−0.0001	0.000363			
PT*PC	SC2 (green)	0.153	0.000352			

^1^ root mean squared error. ^2^ variables with *p*-value < 0.05 were insignificant.

## Data Availability

Data available on request from the corresponding author.
